# Sequence-based comparative study of classical swine fever virus genogroup 2.2 isolate with pestivirus reference strains

**DOI:** 10.14202/vetworld.2015.1059-1062

**Published:** 2015-09-10

**Authors:** Ravi Kumar, Kaushal Kishor Rajak, Tribhuwan Chandra, Dhanavelu Muthuchelvan, Arpit Saxena, Dheeraj Chaudhary, Ajay Kumar, Awadh Bihari Pandey

**Affiliations:** 1 Division of Virology, Indian Veterinary Research Institute, Mukteswar, Nainital, Uttarakhand, India; 2 Department of Biotechnology, Graphic Era University, Dehradun, Uttarakhand, India

**Keywords:** classical swine fever virus, genogroup, genome, pestivirus, phylogenetic tree, sequence

## Abstract

**Aim:**

This study was undertaken with the aim to compare and establish the genetic relatedness between classical swine fever virus (CSFV) genogroup 2.2 isolate and pestivirus reference strains.

**Materials and Methods:**

The available complete genome sequences of CSFV/IND/UK/LAL-290 strain and other pestivirus reference strains were retrieved from GenBank. The complete genome sequence, complete open reading frame, 5’ and 3’ non-coding region (NCR) sequences were analyzed and compared with reference pestiviruses strains. Clustal W model in MegAlign program of Lasergene 6.0 software was used for analysis of genetic heterogeneity. Phylogenetic analysis was carried out using MEGA 6.06 software package.

**Results:**

The complete genome sequence alignment of CSFV/IND/UK/LAL-290 isolate and reference pestivirus strains showed 58.9-72% identities at the nucleotide level and 50.3-76.9% at amino acid level. Sequence homology of 5’ and 3’ NCRs was found to be 64.1-82.3% and 22.9-71.4%, respectively. In phylogenetic analysis, overall tree topology was found similar irrespective of sequences used in this study; however, whole genome phylogeny of pestivirus formed two main clusters, which further distinguished into the monophyletic clade of each pestivirus species. CSFV/IND/UK/LAL-290 isolate placed with the CSFV Eystrup strain in the same clade with close proximity to border disease virus and Aydin strains.

**Conclusion:**

CSFV/IND/UK/LAL-290 exhibited the analogous genomic organization to those of all reference pestivirus strains. Based on sequence identity and phylogenetic analysis, the isolate showed close homology to Aydin/04-TR virus and distantly related to Bungowannah virus.

## Introduction

The genus pestivirus of the family *Flaviviridae* comprised of bovine viral diarrhea virus-1 (BVDV-1), BVDV-2, classical swine fever virus (CSFV), and border disease virus (BDV) [1]. Apart from these four established species, various tentative species also detected *viz*. pestivirus of giraffe [2], BVDV-3 or atypical bovine pestiviruses (“HoBi”-like viruses) [3,4], pronghorn virus [5], Bungowannah virus [6] and Tunisian sheep virus (TSV) (previously termed “Tunisian isolates”), and Turkey sheep virus [7]. Further, BVDV-1, BVDV-2, BDV, and CSFV have been characterized into genotypes, subtypes, and subgenotypes; BVDV-1 and BVDV-2 are having 17 and 3 subtypes, respectively; BDV and CSFV is comprised of 7 and 3 genotypes, respectively, CSFV three genotypes can be further divided into three or four subgenotypes [8].

Pestiviruses have single-stranded, positive-sense RNA genome of approximately 12.3-12.7 kilobases (kb) that contains only one open reading frame (ORF). The ORF translated into a polyprotein about 3,900 amino acids (aa) flanked by highly conserved non-coding regions (NCRs) at 5’nd 3’ends. The ORF encodes a polyprotein that is co- and post-translationally processed into twelve structural and non-structural proteins. These proteins arranged from 5’ to 3’ ends are: N-terminal protein (Npro), capsid protein (C), envelope glycoprotein (Erns, E1 and E2), protein 7 (p7), and the non-structural proteins (NS) NS2, NS3, NS4A, NS4B, NS5A, and NS5B [1].

Molecular epidemiology based on complete nucleotide sequence data has broadened the scope for genetic heterogeneity and phylogenetic grouping of isolates existing in field condition. The genetic relatedness of pestivirus isolates, including their relationship to the type viruses of the species, is one of the important parameters for classification of pestiviruses [9]. During the past decade, much progress on the molecular characterization of pestiviruses has been achieved. Although, few isolates have been sequenced completely, and comparative studies on full genome sequence are scanty [10-13]. Generation of complete genome sequence data will help in better understanding of the molecular biology of any virus both at structural and functional level. There is no report where CSFV has been compared with other members of pestiviruses, although reverse studies are various [8,13-16]. Recently, full genome sequencing of CSFV genogroup 2.2 isolate, CSFV/IND/UK/LAL-290 has been done [17]. To compare CSFV/IND/UK/LAL-290 isolate with other members of pestiviruses, the present study was undertaken.

## Materials and Methods

### Ethical approval

Ethical approval was not required for this type of study as live animal is not used anywhere in this study.

### Sequence Retrieval and Analysis

Sequences used in this study were retrieved from GenBank (http://www.ncbi.nlm.nih.gov/genbank/) on 2 April 2015 (Table-1). Different sequences like the complete genome complete ORF, 5’ and 3’ NCR were aligned with respective sequences of reference pestiviruses strain. Sequence analysis was carried out by Clustal W model in MegAlign program of Lasergene 6.0 software (DNASTAR Inc., Madison, WI, USA).

**Table-1:** Heterogeneity and percent identity of CSFV/IND/UK/LAL290 with reference to pestivirus strains.

Strains	Accession no.	Complete genome (nucleotide) (%)		ORF (%)				NCRs (%)			
	
				Nucleotide		Amino acid		5’ NCR		3’ NCR	
CSFV/IND/UK/LAL290*	KC_851953	12297 bp	-	11697 bp	-	3898 aa	-	373 bp	-	227 bp	-
CSFV/Eystrup**	NC_002657	12301 bp	84.1	11697 bp	83.7	3898 aa	89.7	373 bp	93	231 bp	85
BDV/X818	NC_003679	12333 bp	66.4	11688 bp	67.3	3895 aa	68.8	372 bp	76.1	273 bp	31.2
BVDV1/NADL	NC_001461	12573 bp	66.8	11967 bp	67	3988 aa	67.2	385 bp	67.3	221 bp	42.7
BVDV2/C413	NC_002032	12255 bp	68.1	11694 bp	67.9	3897 aa	69.8	355 bp	74.3	206 bp	58.6
BVDV3/Th/04 KhonKaen	NC_012812	12337 bp	68.1	11700 bp	68	3899 aa	69.7	383 bp	77.5	254 bp	36.1
Pestivirus Giraffe	NC_003678	12602 bp	72	11970 bp	71.6	3989 aa	76.9	382 bp	82.3	250 bp	71.4
Pestivirus Aydin/04TR	NC_018713	12292 bp	71.2	11688 bp	70.8	3895 aa	75.2	277 bp	79.6	227 bp	55.5
Pestivirus pronghorn antelope	NC_024018	12273 bp	58.9	11694 bp	59.6	3897 aa	50.3	369 bp	64.1	210 bp	22.9
Pestivirus Bungowannah	NC_023176	12656 bp	62	11757 bp	61.1	3918 aa	56.4	399 bp	68.8	500 bp	51.4

* CSFV isolate under study

** CSFV reference strain, bp=base pair (nucleotide), aa=amino acid, %=percent identity, BVDV=Bovine viral diarrhea virus, ORF=Open reading frame, NCR=Noncoding region, CSFV=Classical swine fever virus, BDV=Border disease virus

### Phylogenetic Analysis

Phylogenetic analysis was performed using MEGA 6.06 software package [18]. The evolutionary history was inferred using the maximum likelihood method, and the tree topologies were evaluated using 1000 replicates (bootstrap value) of the data set. Best DNA/protein model for construction of phylogenetic tree was selected from model test program integrated with MEGA 6.06 software. Trees based on 5′ and 3′ NCRs sequence were constructed using Kimura 2 (G) and Tamura 3 (G) parameters, respectively. Whereas, trees of the complete genome and complete ORF sequence were constructed by general time reversible parameters having gamma distribution with invariable sites (G+I).

## Results and Discussion

Like other RNA viruses, pestiviruses also exhibit genetic heterogeneity which has been supported by several workers [10-16]. Comparative sequence analysis of CSFV/IND/UK/LAL-290 exhibited analogous genomic organization to those from reference pestivirus strains. CSFV isolate under study showed genome length of 12,297 nucleotides (nt), including a 373-nt 5’ untranslated region (UTR), an 11,697-nt ORF encoding a 3,898-amino-acid-long polyprotein, and a 227-nt 3’ UTR [17]. The average sequence length of complete genome, ORF (both nucleotides and amino acids), 5’ and 3’ NCRs of reference pestivirus strains were found to be 12255-12656 bp, 11688-11970 bp (3895-3989 -aa), 355-399 bp and 206-500 bp, respectively (Table-1). Upon complete genome analysis, BVDV2/C413 strain exhibited the smallest genome (12255 bp); while Bungowannah strain has the largest genome (12656 bp) amongst reference pestivirus strains. BDV/X818 and pestivirus Aydin/04-TR shared the smallest ORF with 3895 aa while, pestivirus Giraffe genome showed the largest ORF with 3989 aa.

The sequence alignment of CSFV/IND/UK/LAL-290 isolate with reference pestivirus strains of complete genome level showed 58.9-72% identities at the nucleotide level and 50.3-76.9% at amino acid level. Further 5’and 3’ NCRs of the isolate showed 64.1-82.3% and 22.9-71.4% sequence identity, respectively as compared to other pestiviruses. The 5’NCR of present CSFV isolate was also found to be highly conserved than 3’ NCR like other pestiviruses. Among the genus of family *Flaviviridae*, pestivirus has relatively long 5’ NCR. The 5’ NCR of the present isolate was most similar to that of other members of CSFV and BVD with regard to length and sequence, which corroborate the finding of Becher *et al*. [13]. The 3’ NCR of CSFV/IND/UK/LAL-290 genome showed considerable heterogeneity in size. This is similar to the earlier report for BDV, BVDV-1, and other CSFV strains. For BVDV-1 strains (NADL and SD-1) and for most CSFV strains, the 3’ end has been reported to be approximately 226 nucleotides long, while those of BVDV-1 strain Osloss and BVDV-2 strain 890 comprised only 185 and 206 nucleotides, respectively [13]. The 3’ NCR of CSFV C-strain consists of 241 nucleotides. Surprisingly, the 3’ NCR of present isolate consisted of 227 nucleotides which were similar to pestivirus Aydin/04-TR.

The percent identity of CSFV/IND/UK/LAL-290 with reference pestivirus strains is summarized in Table-1; wherein it showed close homology with Aydin/04-TR virus and distant relation with Bungowannah virus. When the whole genome of CSFV/IND/UK/LAL-290 isolate compared with other submitted CSFV strains; it showed 82.0-91.1% identities at the nucleotide level and 87.9-92.5% at amino acid level [17]. Comparison with CSFV reference strain Eystrup (genogroup 1.1 virus) revealed 84.1% and 89.7% identity at nucleotide and amino acid level, respectively (Table-1).

**Figure-1:**
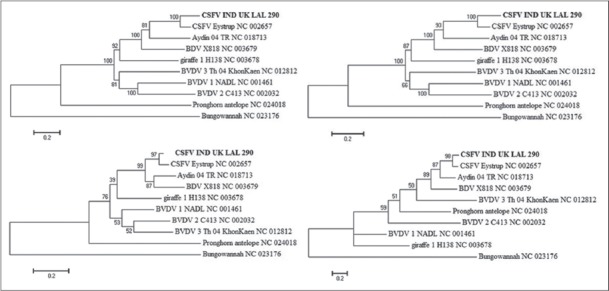
Maximum likelihood (ML) trees depicting phylogenetic relationship of classical swine fever virus/IND/UK/LAL-290 isolate among pestivirus reference strains at, (a) complete genome and (b) open reading frame (ORF) nucleotide sequence level with general time reversible (GTR+G+I) model; (c) 5’ NCR with Kimura 2 (K2+G) model, and (d) 3’ NCR with Tamura 3 (T92+G) model. The bootstrap consensus was inferred from 1,000 replicates to represent the evolutionary history of the *taxa* analyzed, and branch lengths were indicative of genetic distances between the sequences.

Phylogenetic analysis of pestiviruses is generally carried out based on sequences from the 5’ UTR/NCR, Npro and E2 regions [5,19] or, less frequently, the Npro, E2 and NS3 regions [3,6]. The topology or topography of trees based on the complete genome (Figure-1a), complete ORF (Figure-1b) and 5’ NCR (Figure-1c) sequences were very similar to each of them in comparison to 3’ NCR.

The whole genome phylogeny of pestivirus formed two main clusters, which further distinguished into a monophyletic clade of each pestivirus species. CSFV/IND/UK/LAL-290 isolate placed with the CSFV Eystrup strains in the same clade with close proximity to BDV and Aydin strain (Figure-1a). Pestiviruses of bovine (BVDV-1, BVDV-2, and BVDV-3) were clustered separately from CSFV isolates whereas, Bungowannah and pronghorn antelope formed an independent clade. Results of the present study support the findings of Liu *et al*. [20], but clustering of pestivirus of giraffe was found to be in contrary to them. Pestivirus of giraffe was clustered with CSFV and BDV. The plausible reasons for different tree position of pestivirus of the giraffe could be the use of the different genomic region for making the phylogenetic tree. Liu *et al*. [20] used combined gene sequences of 5’ UTR, Npro and E2 regions for the tree; whereas whole genome was considered in the present study, which has given better resolution of the tree.

In 3’ NCR based tree, all pestivirus strains changed their clustering pattern barring strains of the swine (CSFV strains) and ovine (BDV and Aydin strain) origin (Figure-1d). This could be due to high heterogeneity of the region. Study on phylogenetic analysis using this region has not been done so far where all pestiviruses were taken into consideration. To the best of our knowledge, this is the first report of its own kind where attempts were made to analyze all the reference pestiviruses, however, further study is warranted.

Based on sequence analysis using different region and strategies, different workers have proposed their classification of pestiviruses. Analysis of the genetic and antigenic similarities, Becher *et al*. [19] have suggested seven major antigenic groups of novel pestivirus genotype corresponding to BVDV-1, BVDV-2, CSFV, BDV-1, BDV-2, BDV-3, and “giraffe.” Later on, isolation and identification of antelope, Bungowannah, and Tunisian sheep virus lead to a new proposal of classification. A new proposal is presented for the classification of pestiviruses into nine species: BVDV-1, BVDV-2, BVDV-3 (atypical bovine pestiviruses), pestivirus of giraffe, CSFV, BDV, Tunisian sheep virus (Aydin/04-TR), Antelope, and Bungowannah [20]. Findings of this study also proposed a classification of novel pestiviruses into nine species similar to Liu *et al*. [20]; however, clustering pattern in phylogeny was found to be different.

## Conclusion

In the present study, it was observed that CSFV/IND/UK/LAL-290 showed a comparable genomic organization with that of other pestiviruses. The isolate was closely related to Aydin/04-TR virus and distantly with Bungowannah virus. Phylogenetic analysis revealed that BVDV-1, BVDV-2, BVDV-3 sharing a common ancestor, while Bungowannah and pronghorn antelope formed a single separate clade in all the trees. In contrary to the previous findings, pestivirus of giraffe was clustered with CSFV and BDV. Various researchers have classified the pestiviruses in independent manner and availability of the virus sequences. Proposal of nine species in novel pestiviruses also held true in the present study; though, grouping of viruses was different. This study provides a guideline for the classification of newly detected pestiviruses and has a potential application in genetic analysis of other related viruses in future.

## Authors’ Contributions

RK, KKR, and TC conceptualized the work. RK, DM, DC, AS and AK carried out the work and bioinformatics part. KKR, ABD, and RK drafted the manuscript. KKR, DM, ABP, and TC reviewed and edited the manuscript. All authors read the manuscript and approved the final manuscript.

## References

[1] Simmonds P., Becher P., Collet M.S., Gould E.A., Heinz F.X., Meyers G., Monath T., Pletnev A., Rice C.M., Stiansny K., Thiel H.J., Weiner A., Bukhet J., King A.M.Q., Adams M.J., Carstens E.B., Lefkowitz E.J. (2011). Flaviviridae. Virus Taxonomy: Ninth Report of the International Committee on Taxonomy of Viruses.

[2] Avalos-Ramirez R., Orlich M., Thiel H.J., Becher P. (2001). Evidence for the presence of two novel *Pestivirus* species. Virology.

[3] Schirrmeier H., Strebelow G., Depner K., Hoffmann B., Beer M. (2004). Genetic and antigenic characterization of an atypical pestivirus isolate, a putative member of a novel pestivirus species. J. Gen. Virol.

[4] Bauermann F.V., Ridpath J.F., Weiblen R., Flores E.F. (2013). HoBi-like viruses: An emerging group of pestiviruses. J. Vet. Diagn. Investig.

[5] Vilcek S., Ridpath J.F., Van Campen H., Cavender J.L., Warg J. (2005). Characterization of a novel pestivirus originating from a pronghorn antelope. Virus Res.

[6] Kirkland P.D., Frost M.J., Finlaison D.S., King K.R., Ridpath J.F., Gu X. (2007). Identification of a novel virus in pigs –Bungowannah virus: A possible new species of pestivirus. Virus Res.

[7] Oguzoglu T.C., Tan M.T., Toplu N., Demir A.B., Bilge-Dagalp S., Karaoglu T., Ozkul A., Alkan F., Burgu I., Haas L., Greiser-Wilke I. (2009). Border disease virus (BDV) infections of small ruminants in Turkey: A new BDV subgroup?. Vet. Microbiol.

[8] Weber M.N., Streck A.F., Silveira S., Mósena A.C.S., da Silva M.S., Canal C.W. (2015). Homologous recombination in pestiviruses: Identification of three putative novel events between different subtypes/genogroups. Infect. Genet. Evol.

[9] Heinz F.X., Collett M.S., Purcell R.H., Gould E.A., Howard C.R., Houghton M., Moormann R.J.M., Rice C.M., Thiel H.J. (2000). Family *Flaviviridae*.

[10] Vilcek S., Leskova V., Meyer D., Postel A., Becher P. (2014). Molecular characterization of border disease virus strain Aveyron. Vet. Microbiol.

[11] Mao L., Li W., Zhang W., Yang L., Jiang J. (2012). Genome sequence of a novel Hobi-like pestivirus in China. J. Virol.

[12] Gupta P.K., Saini M., Dahiya S.S., Patel C.L., Sonwane A.A., Rai D.V., Pandey K.D. (2011). Molecular characterization of lapinized classical Swine Fever vaccine strain by full-length genome sequencing and analysis. Anim. Biotechnol.

[13] Becher P., Orlich M., Thiel H.J. (1998). Complete genomic sequence of border disease virus, a pestivirus from sheep. J. Virol.

[14] Becher P., Schmeiser S., Oguzoglu T.C., Postel A. (2012). Complete genome sequence of a novel pestivirus from sheep. J. Virol.

[15] Giangaspero M., Harasawa R. (2011). Species characterization in the genus *Pestivirus* according to palindromic nucleotide substitutions in the 5’-untranslated region. J. Virol. Methods.

[16] Postel A., Schmeiser S., Oguzoglu T.C., Indenbirken D., Alawi M., Fischer N., Grundhoff A., Becher P. (2015). Close relationship of ruminant pestiviruses and classical swine fever virus. Emerg. Infect. Dis.

[17] Kumar R., Rajak K.K., Chandra T., Thapliyal A., Muthuchelvan D., Sudhakar S.B., Sharma K., Saxena A., Raut S.D., Singh V.K., Ahmad Z., Kumar A., Chaudhary D., Singh R.K., Pandey A.B. (2014). Whole-genome sequence of a classical swine fever virus isolated from the Uttarakhand state of India. Genome Announc.

[18] Tamura K., Stecher G., Peterson D., Filipski A., Kumar S. (2013). MEGA6: Molecular evolutionary genetics analysis version 6.0. Mol. Biol. Evol.

[19] Becher P., Raminez R.A., Orlich M., Rosales S.C., Konig M., Schweizer M., Stalder H., Schirrmeier H., Thiel H.J. (2003). Genetic and antigenic characterization of novel pestivirus genotypes: Implication for classification. Virology.

[20] Liu L., Xia H., Wahlberg N., Belák S., Baule C. (2009). Phylogeny, classification and evolutionary insights into pestiviruses. Virology.

